# Different roles of brain uroguanylin and brown adipose tissue in males and females

**DOI:** 10.1186/s13293-026-00965-y

**Published:** 2026-08-11

**Authors:** Nikola Habek, Martina Ratko, Milan Kordić, Marina Dobrivojević Radmilović, Siniša Škokić, Vladiana Crljen, Martina Tkalčić, Anton Mažuranić, Pero Bubalo, Petar Škavić, Aleksandra Dugandžić

**Affiliations:** 1 Croatian Institute for Brain Research, School of Medicine, University of Zagreb, Zagreb, Croatia; 2 Centre of Excellence for Basic, Clinical and Translational Neuroscience, School of Medicine, University of Zagreb, Zagreb, Croatia; 3 Department of Physiology, School of Medicine, University of Zagreb, Zagreb, Croatia; 4 MKP Ltd. Zagreb, Zagreb, Croatia; 5 Institute for Forensic Medicine, School of Medicine, University of Zagreb, Zagreb, Croatia

**Keywords:** Estrus vs. diestrus, GLP-1, i.n. vs. i.p. application, Insulin, Uroguanylin in the brain

## Abstract

Obesity and type 2 diabetes mellitus (T2DM) are major burdens on global health today. A novel therapeutic approach could involve increasing postprandial glucose utilization through the activation of brown adipose tissue (BAT). The aim of this study was to determine role of brain uroguanylin (UGN) in sex differences in BAT function. BAT activity was assessed by infrared thermography in wild-type C57Bl/6NCrl mice and UGN KO littermates. The volume of BAT was determined by MRI, while markers of browning were analysed by qPCR. The expression of proUGN in mouse and human brain tissue was determined by ELISA. In the human brain, decreased proUGN expression in subjects with obesity was observed only in the male hypothalamus, BA10, and BA11. The effects of centrally applied UGN depend not only on sex, but also on the phase of the estrous cycle. Sex differences in the postprandial regulation of BAT activity in mice may be caused by differential postprandial regulation of hypothalamic proUGN expression, which was observed only in male mice. In young male mice, intranasal administration of a GLP-1 analogue, as possible regulator of UGN expression in the brain, increased BAT activity and decreased hypothalamic proUGN expression only in males. Although chronic UGN administration also increased postprandial BAT activation in both sexes, increased BAT volume was observed only in male mice. Due to the different physiological and pathophysiological functions of BAT and its activators, the introduction of brain BAT activators as a therapy for obesity and T2DM must take sex into account.

**Supplementary Information:**

The online version contains supplementary material available at https://doi.org/10.1186/s13293-026-00965-y.

## Abstract (plain language summary)

Obesity and type 2 diabetes are major health problems. A new way to treat them is by activating “brown fat” (a type of fat that burns energy to create heat) to improve blood sugar control after meals. Specifically, researchers studied how a brain hormone called uroguanylin (UGN) affects brown fat, and how this process differs between men and women. In human brains, source of UGN (proUGN) is decreased only in men with obesity, which was not observed in female brains. In mice, activating brown fat after eating depended on the sex. The mechanism that links eating to brown fat activation via UGN was shown only in male mice. While chronic UGN treatment helped both sexes, only in males there was an increase in the total amount of brown fat. When GLP-1 analogue was applied in the brain, it increased brown fat activity in both male and female mice, but affected brain UGN levels only in male mice suggesting completely different mechanism of GLP-1 function in the brain. Targeting brain-activated brown fat to fight obesity and diabetes may work differently in men and women. Future therapies need to take sex differences into account to be effective.

**Supplementary Information:**

The online version contains supplementary material available at https://doi.org/10.1186/s13293-026-00965-y.

## Highlights

Expression of the BAT-regulating hormone UGN in the mouse and human brain is sex-dependent.

Regulation of BAT activity by UGN depends on sex and the phase of the estrous cycle.

The role of brain UGN in obesity differs between the male and female human brain.

**Supplementary Information:**

The online version contains supplementary material available at https://doi.org/10.1186/s13293-026-00965-y.

## Introduction

Brown adipose tissue (BAT), in contrast to white adipose tissue (WAT), does not store energy, but rather expends it. In both humans and rodents, fasting blood glucose concentration is negatively correlated with BAT activity [1, 2], suggesting its role in the development of obesity and type 2 diabetes mellitus (T2DM).

There are two physiological conditions under which BAT activation occurs: Cold exposure induces BAT activation, which improves insulin sensitivity in humans [3], and this activity is decreased in older individuals and in people with obesity and T2DM [4–8]. The accumulation of ^18^F-FDG (Fluorodeoxyglucose) in BAT is lower in men than in women. This sex difference is more pronounced in younger participants. Decreased ovarian function leads to a reduction in BAT metabolic activity. The age-related decline is more pronounced in male subjects [5, 6, 9]. After a meal, BAT activation leads to increased postprandial glucose and fatty acid consumption, resulting in heat production [10–12], which is dependent on sex, age, and body mass index (BMI) [2]. Glucose uptake in BAT after a meal increases in lean male subjects, and diet-induced thermogenesis (DIT) is higher in BAT-positive male subjects detected by PET-CT. However, DIT is not BMI-dependent in female participants [10, 11, 13].

Uroguanylin (UGN) belongs to the guanylin peptide family of natriuretic peptides. Systemic UGN, originating from the intestine after a meal, induces lipolysis and diuresis [14]. The concentration of proUGN in plasma is decreased in people with obesity [15] and in obese mice [16]. Its receptor, guanylate cyclase C (GC-C), is expressed in many organs, including several brain regions in both sexes, such as the human prefrontal cortex (PFC), hypothalamus, substantia nigra, and cerebellar cortex [17]. In the human PFC, GC-C expression decreases after feeding in male Brodmann areas (BA) 11 and 10. In female BA11, no correlation between feeding and GC-C expression has been found, whereas in BA10, GC-C expression is even higher after a meal. This suggests sex differences in the regulation of GC-C expression in BA11 and BA10 [17, 18]. In the female hypothalamus, GC-C expression is lower than in the male hypothalamus under fasting conditions, suggesting that women may have reduced satiety and BAT activation responses to GC-C agonists after a meal [17].

In male rodents, GC-C has been found in neurons of the cerebral cortex, amygdala, midbrain, and cerebellum [19–21], as well as in the hypothalamus, especially in the ARC (arcuate nucleus) and ventral premammillary nucleus [2, 22]. In the female hypothalamus, there is no postprandial regulation of GC-C expression, and GC-C is not involved in BAT growth, which has been detected in male mice. Furthermore, hypothalamic GC-C is less expressed during estrus (follicular phase) than during diestrus (luteal phase), which suggests differences in GC-C agonist function during the female estrous cycle [20]. GC-C in the majority of brain regions can be activated only by brain-derived UGN [23].

In our previous study, we found that proUGN expression in the male human PFC is decreases after a meal, and that this effect is lost in obesity. In the male human hypothalamus, proUGN expression is lower in obesity, suggesting reduced availability of UGN precursors [23]. GC-C activation by UGN applied into the brain ventricles of diet-induced obese male mice decreases food intake, leads to weight loss, and increases browning of WAT via sympathetic activation [24]. Similarly, centrally applied UGN and its analogue linaclotide induce acute and chronic activation of BAT in male mice [23]. Accordingly, when the GC-C signaling pathway is absent, BAT markers are expressed at lower levels in male GC-C KO mice [2].

The aim of this study was to determine sex/gender differences in the physiological and pathological effects of brain UGN and postprandial BAT activation. In the human brain, changes in proUGN expression in subjects with obesity are gender-dependent. Postprandial changes in proUGN expression, BAT activity, and their regulation are dependent on sex and on the phase of the estrous cycle. Since BAT is regulated in the brain by UGN, leptin, and glucagon-like peptide-1 (GLP-1) [25], the introduction of brain BAT activators as a therapy for obesity and T2DM must take sex and the phase of the estrous cycle into account.

## Methods

### Animals

In this study, 6-month-old male and female wild-type (WT) C57Bl/6NCrl mice and UGN KO littermates were used [23]. Mice were fed standard rodent chow *ad libitum* and had free access to water. They were housed under a 12 h light/12 h dark cycle at 23 °C and 50–75% humidity. All experiments were approved by the Ethics Committee of the University of Zagreb School of Medicine (641-01/18 − 04/01). Experimental procedures were performed in accordance with the Ethical Codex of the Croatian Society for Laboratory Animal Science and the ARRIVE guidelines. For anaesthesia, animals were anesthetized by intraperitoneal (i.p.) administration of ketamine/xylazine (80–100 mg/kg and 10–12.5 mg/kg, respectively).

### Human brain tissue

Human brain tissue was collected from 34 deceased individuals (21 males, 53 ± 3 years; 13 females, 64 ± 6 years; Additional Table 1) with a postmortem delay of less than 24 h and without neuropathological abnormalities in brain structures. Obesity was assessed by forensic physicians during autopsy. Experimental procedures were approved by the Ethics Committee of the University of Zagreb School of Medicine (641-01/18 − 02/01). Samples were collected from the arcuate nucleus, substantia nigra (midbrain), medial cerebellar cortex (vermis), and Brodmann areas (BA) 9, 10-O, 10-M, 11, and 32 of the prefrontal cortices (PFC), and were stored at − 80 °C until further use. Sample collection was approved by the deceased individuals’ next of kin through signed informed consent. All researchers involved in this study protected the personal information and identity of the deceased. This information was not and will not be disclosed to third parties.

### Hormone administration

Peptides were administered either intranasally (i.n.) or intraperitoneally (i.p.). After 4 h of fasting, UGN was administered i.n. at a dose of 10 µg/animal or i.p. at a dose of 50 µg/animal. BAT activity was measured before administration and at 15 and 30 min, and 1 and 2 h thereafter [26]. In separate experiments, interscapular BAT activity was measured before and 30 min after i.n. administration of insulin (10 mU/animal in saline, total volume 5 µL) or a GLP-1 analogue (30 µg/animal in saline, total volume 5 µL). Animals were anesthetized as described above. Blood was collected into heparinized tubes, and plasma was stored at − 20 °C until analysis. After decapitation, the hypothalamus was isolated, rapidly frozen in liquid nitrogen, and stored at − 20 °C until analysis.

### Determination of the estrous cycle phase

In female mice, the phase of the estrous cycle was determined on the evening before the experiment and rechecked in the morning immediately before the experiment, as previously described [27]. A cotton swab soaked in sterile saline solution at room temperature was inserted into the mouse vagina. After gently scraping the vaginal wall with the swab, the attached cells were spread onto a glass slide and air-dried. Cells were stained with 1% cresyl violet acetate for 1 min and rinsed three times with distilled water. Under a light microscope, the phase of the estrous cycle was determined based on the numbers of leukocytes, nucleated epithelial cells, and cornified epithelial cells observed in the smear.

### Mouse brain isolation

To determine meal-induced changes in proUGN expression animals were fasted overnight and sacrificed by cervical dislocation before feeding and at 30 and 120 min after a meal. Plasma and brain samples were collected and rapidly frozen in liquid nitrogen and stored at − 20 °C until analysis. All efforts were made to minimize animal suffering and reduce the number of animals.

### Chronic UGN administration

The postprandial increase in BAT activity, as well as basal BAT and WAT volume, was determined in male and female WT mice by infrared thermography and magnetic resonance (MR) imaging. Animals were randomly assigned to two groups (*n* = 6). In the control group, saline was administered intranasally twice daily, in the morning (8a.m.; 5 µL) and in the afternoon (4p.m.; 5 µL), for 2 weeks. The experimental group received UGN at the same time points (5 µg/animal in 5 µL saline per administration). The final daily UGN dose was 10 µg/animal [2]. After 2 weeks of i.n. administration, the volumes of interscapular BAT and WAT and BAT activation after a meal were reassessed, after which the animals were anesthetized and sacrificed by cervical dislocation. Interscapular BAT and epigonadal white adipose tissue (eWAT) were isolated, rapidly frozen in liquid nitrogen, and stored at − 80 °C until analysis. Samples were analysed by Quantitative real-time PCR (qPCR) and Enzyme-linked immunosorbent assay (ELISA).

### Quantitative real-time PCR (qPCR)

Total RNA was isolated from BAT and WAT tissue using TRI Reagent^®^ Solution (Thermo Fisher Scientific, Waltham, MA, USA). cDNA was synthesized from 1 µg of total RNA using the GoScript Reverse Transcription System (Promega, Madison, WI, USA). cDNA (1 µL) was used for the detection of uncoupling protein 1 (*Ucp1*, Mm01244861_m1, Thermo Fisher Scientific, Waltham, MA, USA), positive regulatory domain zinc finger protein 16 (*Prdm16*, Mm00712556_m1, Thermo Fisher Scientific), and peroxisome proliferator-activated receptor gamma coactivator 1-alpha (*Pgc1A*, Mm01208835_m1, Thermo Fisher Scientific). Hypoxanthine guanine phosphoribosyl transferase (*Hprt*, Mm03024075_m1, Thermo Fisher Scientific) was used as the housekeeping gene for normalization of mRNA expression in BAT and WAT tissue. All qPCR reactions were performed using a 7500 Real-Time PCR System (Thermo Fisher Scientific).

### Enzyme-linked immunosorbent assay (ELISA)

An enzyme-linked immunosorbent assay (ELISA) was used to determine proUGN expression in mouse and human brain tissue and its concentration in plasma. Frozen human brain samples were thawed on ice and washed in ice-cold PBS. The following PFC regions were sampled: BA9, BA10-O, BA10-M, BA11, and BA32, as previously described [23]. Mouse and human brain tissue samples were homogenized in ice-cold PBS (1 g tissue/9 mL PBS) using an ultrasonic processor (Q55 Sonicator^®^, QSonica Sonicators, Newton, CT, USA), and the homogenate was centrifuged (5 min, 5,000 × g, 4 °C; Eppendorf Centrifuge 5415 R, Hamburg, Germany). For human samples, E2738Hu (Bioassay Technology Laboratory, Birmingham, United Kingdom) was used, whereas for mouse samples, MBS912299 (MyBioSource, San Diego, CA, USA) was used, according to the manufacturers’ instructions.

### MR imaging and data acquisition

MRI was performed using a Bruker BioSpec 70/20 USR system with ParaVision 7.0 software (Bruker BioSpin, Germany) in a Tx/Rx configuration, with an 86 mm volume coil (MT0381, Bruker BioSpin, Germany) for transmission (Tx) and a 2-element mouse brain surface coil (MT0042, Bruker BioSpin, Germany) for reception (Rx), as previously described [23].

Mice were anesthetized with 4% isoflurane (Isoflurane, Abbott, UK) in a 70/30% N₂/O₂ mixture and maintained with 1–1.5% isoflurane in the same gas mixture. Respiratory rate was maintained at 80–100 breaths/min, while body temperature was monitored using a rectal temperature probe and maintained at 37 ± 0.2 °C. Animals were placed in the Bruker MRI mouse cradle, and the receive coil was positioned over the interscapular region. Reference anatomical images were acquired using a turbo spin-echo sequence (TurboRARE: repetition time [TR]/echo time [TE] 1000/10 ms; RARE factor 4). Fifteen slices were acquired at an in-plane isotropic resolution of 140 μm. Slice thickness and interslice gap were 1 mm and 0.5 mm, respectively. The reference scan duration was 25 s.

For fat fraction measurements, two identical high-resolution turbo spin-echo proton density-weighted (PDw) sequences were used (TR = 5000 ms, TE = 7.5 ms, echo spacing = 3.75 ms, RARE = 4, 21 slices, slice thickness = 0.7 mm, interslice gap = 0.3 mm, in-plane isotropic resolution = 100 μm, matrix size = 300 px × 110 px, FOV = 30 mm × 11 mm, 3 averages, receive bandwidth = 210 kHz). To measure the water-only signal, the high-resolution scan was performed with the fat suppression module switched on (PDw_FS scan). Scan duration was 6 min 45 s.

Fat fraction (FF) maps were calculated from PDw and PDw_FS images using FIJI/ImageJ software v1.53f (National Institutes of Health, Bethesda, USA) via a semi-automated procedure with an in-house-developed macro tool. First, the macro reduced the user-defined interscapular ROI to tissue voxels only by thresholding. Next, the proton density fat fraction map was computed by dividing the fat-only image by the fat+water image, using the expression FF = SI_fat/SI_fat+water. This yielded a voxel-wise fat fraction map ranging from 0 to 1. In this expression, SI_fat represents the calculated signal intensity of fat molecules only (SI_fat = SI_fat+water − SI_water = PDw − PDw_FS). After the fat fraction map was calculated, voxels were classified as BAT or WAT based on previously defined thresholds (BAT: 0.2 < FF < 0.7; WAT: FF > 0.7). These thresholds were determined from ex vivo scans of excised BAT and WAT samples [23]. Finally, automatic volumetric analysis, including sample-wise averages and variances, was performed using combined variance formulas that take into account differences in ROI size between slices and samples [28–31].

### Infrared thermography

BAT activation was measured before and after a meal, during which food intake (pellet weight) and body weight were also recorded, as well as after i.n. and i.p. administration of UGN and after i.n. administration of insulin or a GLP-1 analogue. On the day before measurement, the interscapular region was shaved and the phase of the estrous cycle was determined in female mice. After confirmation of the appropriate estrous cycle phase, interscapular BAT activity was measured using a thermal camera (FLIR T-650sc, FLIR Systems, Wilsonville, OR, USA). The investigator was blinded to the experimental groups during image acquisition and analysis. BAT heat energy output was calculated using the Stefan–Boltzmann law, as previously described [2, 23, 32].

### Statistical analysis

Standard parametric analyses were performed according to the number and type of variables. P values < 0.05 were considered statistically significant. Correlations between variables were assessed using Pearson’s correlation test. Dunnett’s or Tukey’s post hoc test was used when appropriate. Results are presented as mean ± SEM or as median with interquartile range. All statistical analyses were performed using GraphPad InStat (GraphPad Software, San Diego, CA, USA) or the Two-Way ANOVA calculator [33].

## Results

### Different role of brain uroguanylin in postprandial regulation of brown adipose tissue activity

**Fig. 1:**
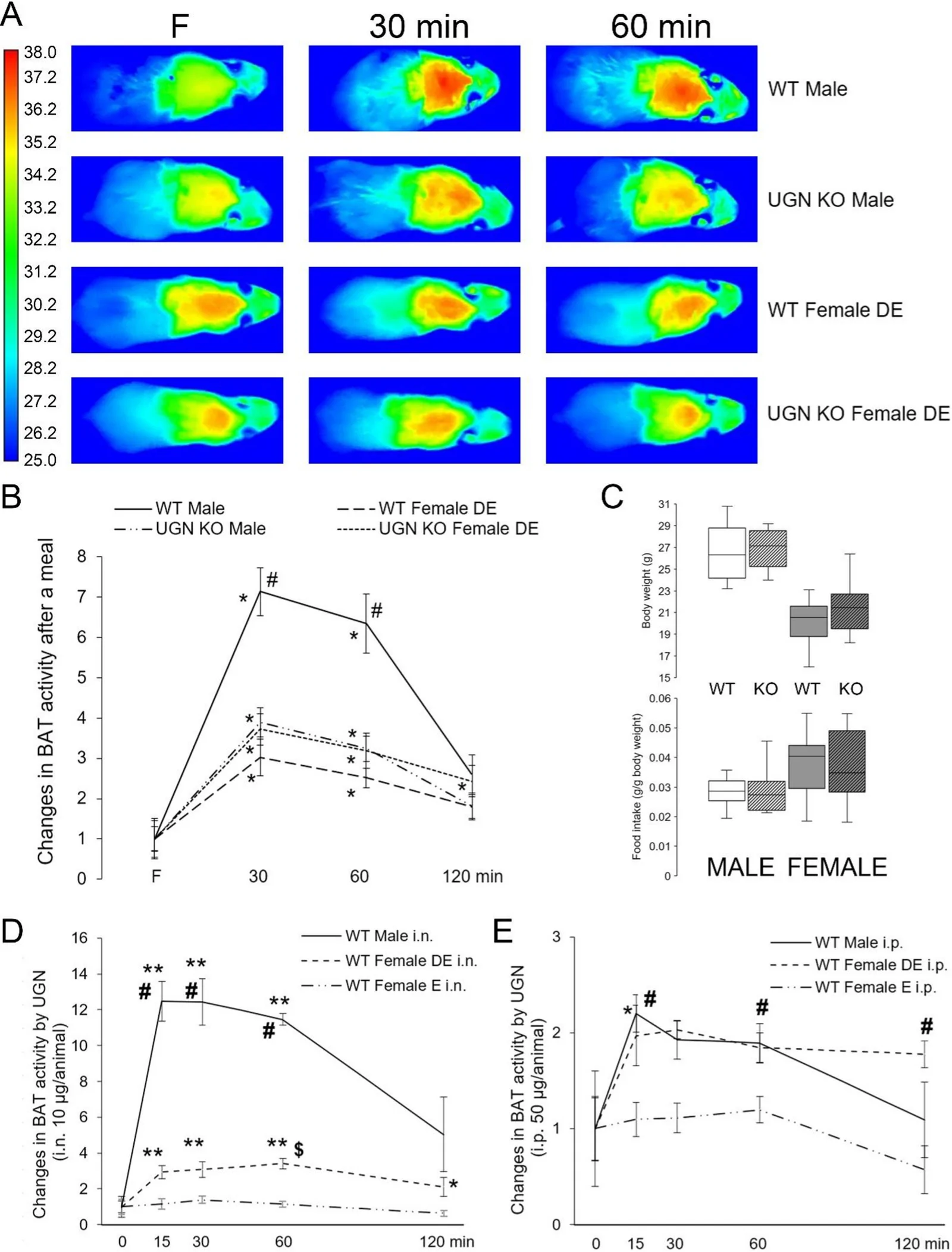
Effects of brain uroguanylin on brown adipose tissue function in male and female mice. (**A**) Thermal images are showing that postprandial BAT activation was lower in male UGN KO mice when compared to WT male mice. Effects of missing UGN on postprandial BAT regulation was not shown in female mice in diestrus (DE). (**B**) Graphical summery of postprandial BAT activation. * *p* < 0.01 compare to fasting (F) (WT male: *p* < 0.0001, F(3,44) = 28.08, UGN KO male: *p* < 0.0001, F(3,44) = 30.85, WT female: *p* = 0.0001, F(3,44) = 9.32, UGN KO female: *p* < 0.0001, F(3,44) = 16.57, paired One Way ANOVA and Dunnett test), # *p* < 0,001 at the same time point (30 min: gender: *p* = 0.000022, F = 22.58, η^2^ = 0.34; genotype: *p* = 0.008972, F = 7.48, η^2^ = 0.15, 60 min: gender: *p* = 0.0001734, F = 16.84, η^2^ = 0.28; genotype: *p* = 0.01382, F = 6.58, η^2^ = 0.13, Two-Way ANOVA), *n* = 12. (**C**) Animals were fed with standard rodent chow with no statistically significant differences in body weight between genotypes and food intake between male and female mice and different genotypes (results are presented as median with interquartile range). (**D**) Acute regulation of BAT activity by intranasally applied UGN (i.n. 10 µg/animal) increased BAT activity the highest in male mice but was completely missing in female mice in estrus (# *p* < 0,001 compared to both group of females, $ *p* < 0,001 compared to females in estrus, One-Way ANOVA, 15 min: *p* < 0.0001, F(2,10) = 77.145; 30 min: *p* < 0.0001, F(2,10) = 56.024; 60 min: *p* < 0.0001, F(2,10) = 411.56; 120 min: *p* < 0.0864, F(2,10) = 3.254; Dunnett Multiple comparisons test; * *p* < 0.05 ** *p* < 0.01 compare to control (0 min), paired One-Way ANOVA, Male: *p* < 0.0001, F(4,15) = 18.909; Female DE: *p* = 0.0001, F(4,15) = 14.836; Female E: *p* = 0.0519, F(4,15) = 3.214, Dunnett Multiple comparisons test). (**E**) Intraperitoneal (i.p.) application (50 µg/animal) activated BAT in male mice while there is no activation in female (*n* = 4, * *p* < 0.05 compare to control (0 min), paired One-Way ANOVA, *p* = 0.0226, F(4,15) = 4.256, Dunnett Multiple comparisons test; # *p* < 0,05 compared to females in estrus, One-Way ANOVA, 15 min: *p* = 0.022, F(2,10) = 5.989; 60 min: *p* = 0.030, F(2,10) = 5.336; 120 min: *p* = 0.041, F(2,10) = 4.674, Dunnett Multiple comparisons test). In male mice, i.n. administration of UGN increased BAT activity more than fivefold compared with i.p. administration (male: 15 min: i.n.=12.5 ± 1.1, i.p.=2.2 ± 0.2, *p* < 0.0001, t(6) = 9.213; 30 min: i.n.=12.4 ± 1.3, i.p.=1.9 ± 0.2, *p* = 0.0002, t(6) = 7.983; 60 min: i.n.=11.5 ± 0.3, i.p.=1.9 ± 0.2, *p* < 0.0001, t(6) = 26.626; 120 min: i.n.=5.0 ± 2.1, i.p.=1.1 ± 0.4, *p* = 0.1179, t(6) = 1.824; Student’s t-test, *n* = 4). In female WT mice in diestrus, i.n. administration only slightly increased BAT activity, whereas no significant BAT activation was observed after i.p. administration (female, diestrus: 15 min: i.n.=2.9 ± 0.4, i.p.=2.0 ± 0.3, *p* = 0.0114, t(6) = 3.600; 30 min: i.n.=3.1 ± 0.4, i.p.=2.0 ± 0.1, *p* = 0.0018, t(6) = 5.336; 60 min: i.n.=3.4 ± 0.3, i.p.=1.8 ± 0.2, *p* = 0.0001, t(6) = 8.875; 120 min: i.n.=2.1 ± 0.5, i.p.=1.8 ± 0.1, *p* = 0.2839, t(6) = 1.177; Student’s t-test, *n* = 4). Neither i.n. nor i.p. administration induced BAT activation in female mice in estrus (female, estrus: 15 min: i.n.=1.2 ± 0.3, i.p.=1.1 ± 0.2, *p* = 0.5992, t(6) = 0.555; 30 min: i.n.=1.4 ± 0.2, i.p.=1.1 ± 0.2, *p* = 0.0781, t(6) = 2.121; 60 min: i.n.=1.2 ± 0.2, i.p.=1.2 ± 0.1, *p* > 0.9999, t(6) = 0.000; 120 min: i.n.=0.6 ± 0.2, i.p.=0.6 ± 0.3, *p* > 0.9999, t(6) = 0.000; Student’s t-test, *n* = 4). Results are presented as means ± SEM. BAT: brown adipose tissue; DE: diestrus; E: estrus; UGN: uroguanylin; UGN KO: UGN knock out littermates

BAT activation after a meal was UGN-dependent only in WT male mice, whereas no difference in postprandial BAT activation was observed between male UGN KO mice, female WT mice, or female UGN KO mice in diestrus (Fig. 1A-original thermal images, Fig. 1B- summarized results). Animals were fed standard rodent chow, and no differences in body weight or food intake were observed between genotypes (Fig. 1C).

To determine whether brain UGN is responsible for the sex difference shown in Fig. 1A and B, UGN was applied in male mice and female mice in estrus and diestrus intranasally (i.n.) or intraperitoneally (i.p.). When UGN was applied i.n., it was expected to be absorbed predominantly into the cerebrospinal fluid (CSF). UGN was administered i.n. at a dose of 10 µg/mouse and i.p. at a fivefold higher dose of 50 µg/mouse. In male mice, i.n. administration of UGN increased BAT activity more than fivefold compared with i.p. administration (Fig. 1D, E). In female WT mice in diestrus, i.n. administration only slightly increased BAT activity, whereas no significant BAT activation was observed after i.p. administration (Fig. 1D, E). In contrast, neither i.n. nor i.p. administration induced BAT activation in female mice in estrus (Fig. 1D, E).

### Differences in plasma proUGN concentrations and hypothalamic expression after a meal are sex-dependent

**Fig. 2:**
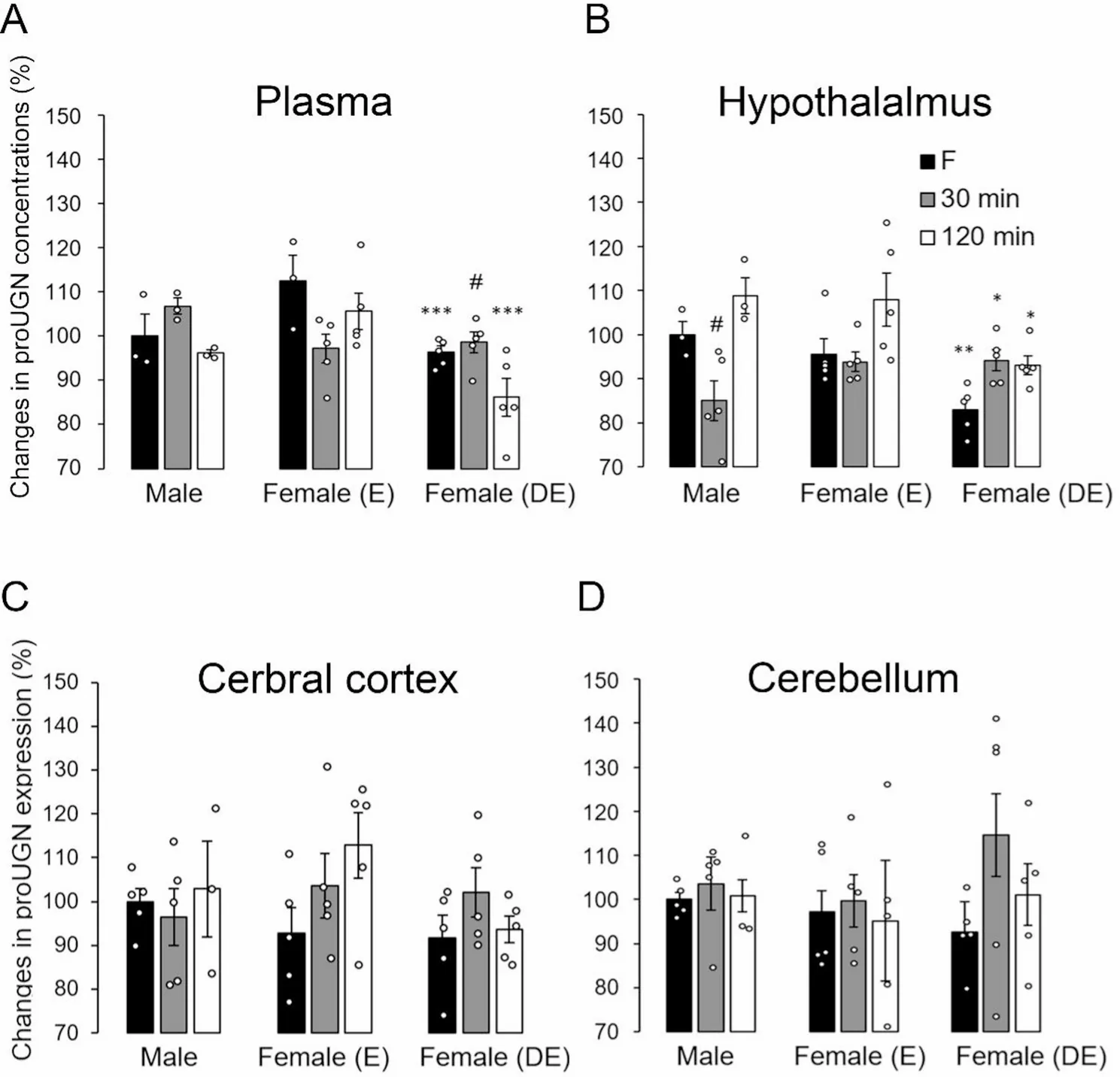
Sex and phase of the estrous cycle differences in postprandial proUGN expression in the mouse brain. Prouroguanylin (proUGN) concertation in plasma (**A**) and expression in hypothalamus (**B**) were postprandially differently regulated in male and female animals in estrus (E) or diestrus (DE) (unpaired One-Way ANOVA, Tukey-Kramer Multiple Comparison test, *n* = 3–5). There were no observed differences in proUGN expression in cerebral cortex (**C**) or cerebellum (**D**). Results are presented as means ± SEM. * *p* < 0.05 compare to fasting (F) (*p* = 0.0085, F(2, 12) = 7.274), ** *p* < 0.01 compared to male mice and female mice in estrus (*p* = 0.0083, F(2, 10) = 8.023), *** *p* < 0.05 compare to female animals in estrus (Fasting: *p* = 0.0358, F(2, 8) = 5.123; 120 min: *p* = 0.0148, F(2, 10) = 6.611) and # *p* < 0.05 compare to 120 min (Hypothalamus: *p* = 0.0084, F(2, 8) = 8.005; Plasma: *p* = 0.0249, F(2, 12) = 5.101)

To determine if sex differences in postprandial BAT activation via UGN is plasma or brain UGN dependent, we determined changes of UGN precursor proUGN in plasma and different brain regions of male mice and female mice in estrus and diestrus. After a meal, changes in plasma proUGN concentrations followed different patterns among the groups. Under fasting conditions and at 120 min after a meal, lower concentrations were detected in female mice in diestrus than in females in estrus (Fig. 2A). Although the pattern of plasma proUGN concentration was similar under fasting conditions and at 120 min after a meal, at 30 min after a meal plasma proUGN was higher in male mice (*p* = 0.0056, t(4) = 5.416, *n* = 3), whereas it was lower in female mice in estrus (*p* = 0.0454, t(6) = 2.518, *n* = 3–5). In female mice in diestrus, plasma proUGN concentrations were lower at 120 min after a meal (*p* = 0.0099, t(4) = 4.620, *n* = 5; Fig. 2A).

In the hypothalamus, postprandial changes in proUGN expression did not parallel the changes observed in plasma concentrations. ProUGN expression was similar under fasting conditions and at 120 min after a meal, except that female mice in diestrus had lower hypothalamic proUGN expression than male mice and female mice in estrus (*p* = 0.0085, t(6) = 3.845). This expression increased at 30 and 120 min after a meal. In male mice, hypothalamic proUGN expression was lower at 30 min after a meal (Fig. 2B).

In other regions of the mouse brain, no statistically significant differences in postprandial proUGN expression were observed in the cerebral cortex or cerebellum between male mice and female mice in estrus or diestrus (Fig. 2C, D).

### Gender-dependent changes in proUGN expression in the brains of overweight subjects

Since physiological role of brain UGN in postprandial BAT regulation was sex-dependent, to established possible role in development of overweight and/or obesity in human population, proUGN expression, as source of UGN, was determined in different brain regions of male and female deceased persons with normal body wight or overweight.

**Fig. 3:**
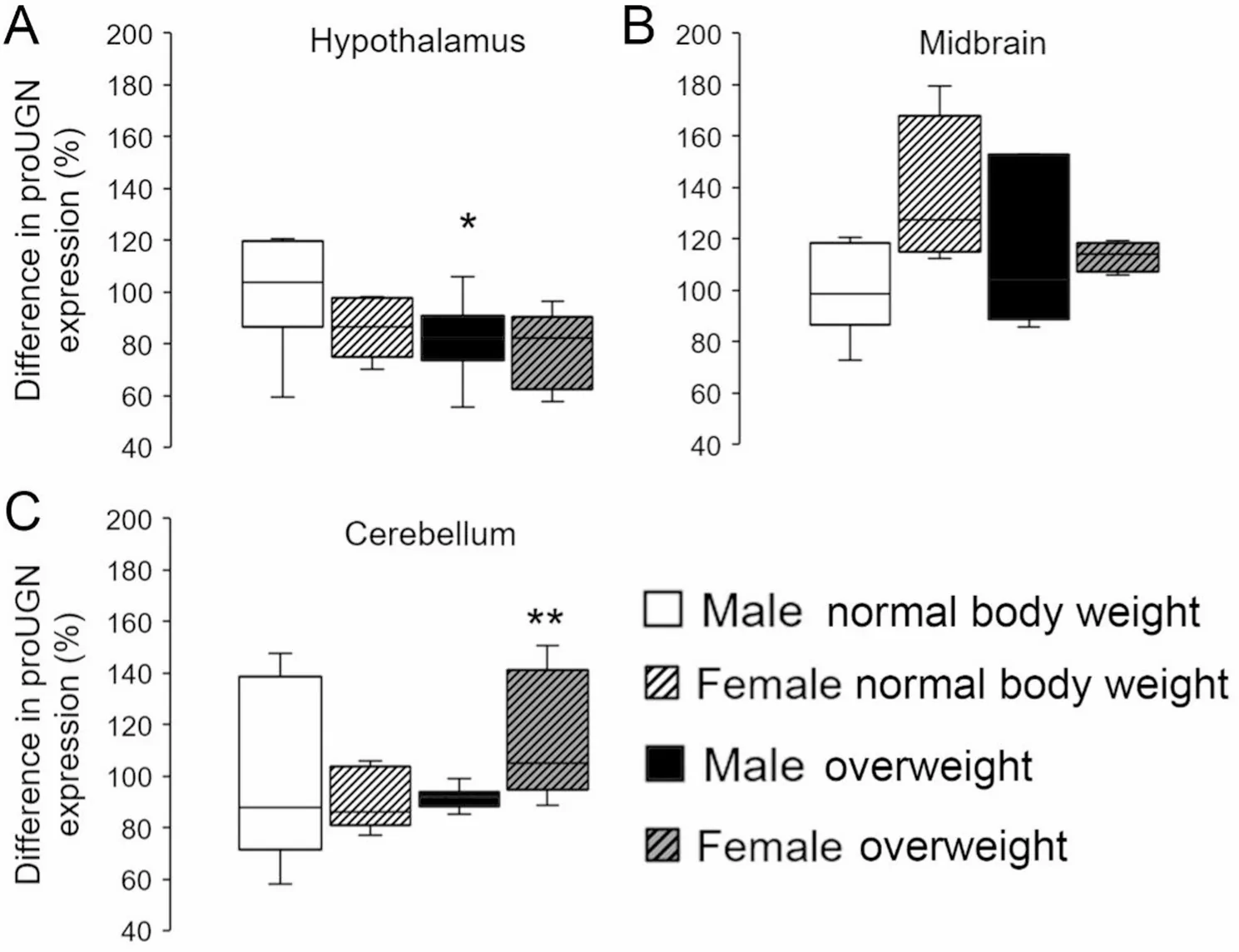
Changes of proUGN expression in obesity is gender dependent. (**A**) ProUGN was less expressed in hypothalamus of male overweight subjects compared to male subjects with healthy body weight. * *p* < 0.05 compare to male controls (*p* = 0.031, F = 5.24, η^2^ = 0.17, Two-Way ANOVA). This was not observed in females. (**B**) In midbrain (substantia nigra) there was no statistically significant difference in proUGN expression. (**C**) In cerebellar cortex (vermis), proUGN expression in overweight female was higher them proUGN expression in female with normal body weight and male overweight subjects (** *p* < 0.05, Two-Way ANOVA for both body weight and gender, *p* = 0.03728, F = 4.7503, η^2^ = 0.14, *n* = 4–11). BMI: body mass index; proUGN – prouroguanylin

In the hypothalamus, sex-dependent differences were observed between overweight subjects and subjects with normal body weight. In the hypothalamus of male overweight subjects, proUGN expression was lower than in subjects with normal body weight. This difference was not observed in the female hypothalamus (Fig. 3A). In the hypothalamus of female subjects, there was no statistically significant correlation between age and proUGN expression in either subjects with normal body weight or overweight subjects (*r* = − 0.618, *p* = 0.191; *r* = − 0.250, *p* = 0.633, respectively).

Because the presence and function of the UGN receptor GC-C in the substantia nigra and cerebellum have been known for some time, we examined whether proUGN expression differed in these brain regions between male and female subjects. In the substantia nigra, proUGN expression in female subjects with normal body weight was 40% higher than in males; however, this difference was not statistically significant (two-way ANOVA, *p* = 0.1215, F = 2.678, η²=0.14). In the cerebellar cortex, more specifically in the human vermis, proUGN expression in female overweight subjects was 25% higher (two-way ANOVA for body weight and sex, *p* = 0.03728, F = 4.7503, η²=0.14) than in female subjects with normal body weight (*p* = 0.0372, t(11) = 2.37) and male subjects with obesity (*p* = 0.0125, t(14) = 2.86; Fig. 3C).

In the cerebellar cortex of male subjects with normal body weight, proUGN expression decreased with age (*r* = − 0.669, *p* = 0.034), whereas this correlation was lost in overweight subjects (*r* = − 0.316, *p* = 0.343). In the female cerebellar cortex, no statistically significant correlation was observed (normal body weight: *r* = − 0.583, *p* = 0.225; overweight: *r* = − 0.251, *p* = 0.587). Thus, regulation of UGN expression differs across specific brain regions, and the effects of increased body weight differ between the male and female brain.

**Fig. 4:**
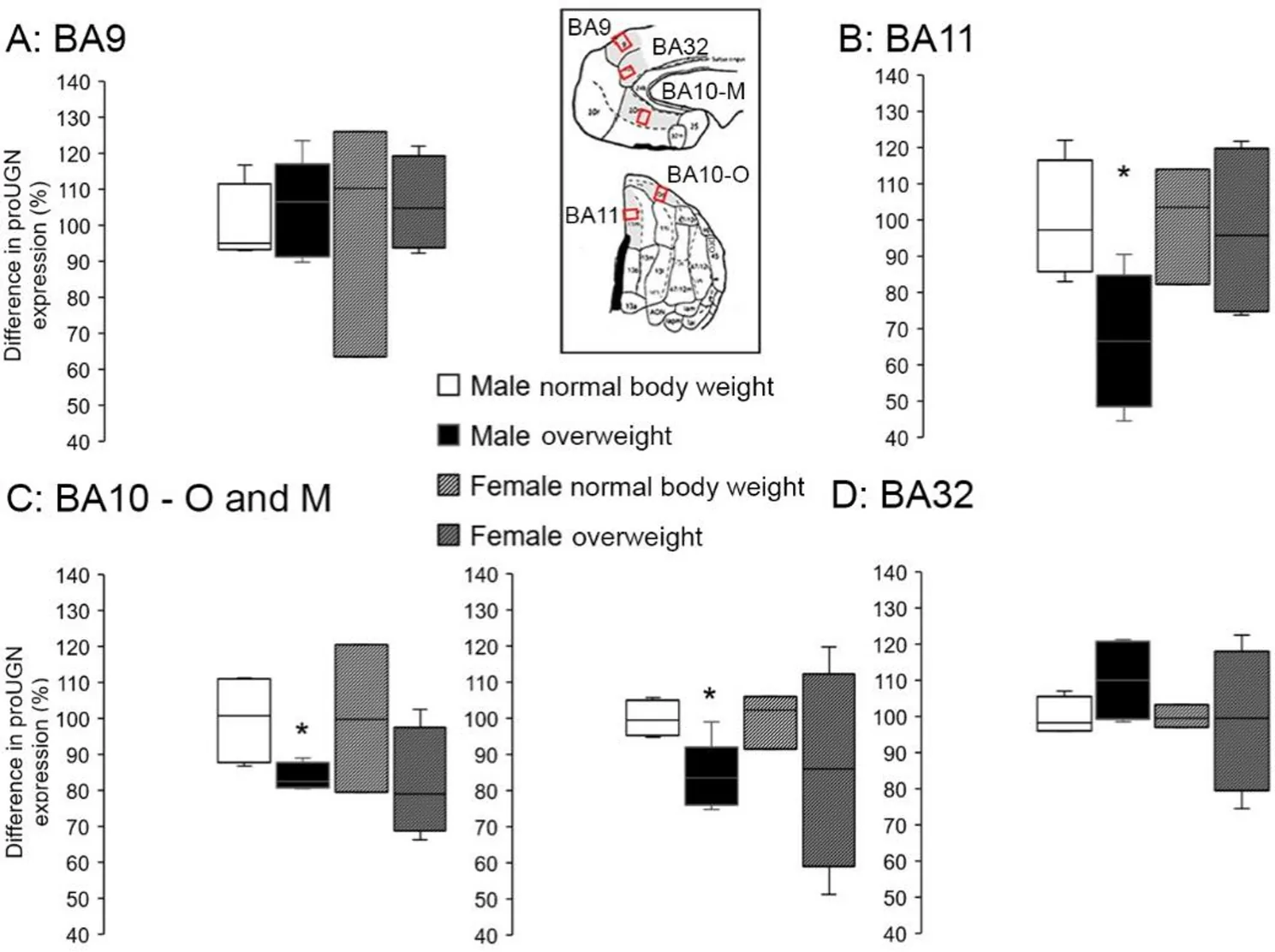
Changes of proUGN expression in human prefrontal cortex in overweight subjects is gender dependent. ProUGN was less expressed in BA10 (**C**, -O and -M) and BA11 (**B**) of male overweight subjects compared to subjects with healthy body weight. This was not observed in females. In both sexes there was no difference in BA9 (**A**) and BA32 (**D**). Results are presented as median with interquartile range. * *p* < 0.05 compare to male subjects with normal body weight, (BA10-O: *p* = 0.03, t(7) = 2.708; BA10-M: *p* = 0.02, t(7) = 3.016; BA11: *p* = 0.03, t(7) = 2.787, unpaired Student’s *t*-test, *n* = 3–6). Schema represents the anatomical location of the sampled tissue. BA: Brodmann area; -O: orbital; -M: medial; BMI: body mass index; proUGN – prouroguanylin

Furthermore, as in the hypothalamus, lower proUGN expression was observed only in male overweight subjects compared with subjects with normal body weight in BA10-O, BA10-M, and BA11 (Fig. 4C, D), whereas no differences were observed in BA9 and BA32 (Fig. 4A, D). These results suggest reduced UGN function in BA10 and BA11 in overweight subjects, but only in males.

### Chronic effects of UGN in male and female mice

To establish if chronic i.n. application of UGN could be used to increase BAT activity, followed by glucose and fatty acids utilisation, in treatment of obesity and T2DM, UGN was applied i.n. daily for 14 days. Experimental groups received i.n. UGN (5 µg/mouse in 5µL) twice daily, whereas control groups received 5µL saline (vehicle). After 4 days of acclimatization, food and water intake, as well as fecal and urine output, were measured (Additional Fig. 1 and additional methods). In WT mice treated with UGN, no statistically significant difference in food or water intake was observed between male and female mice when results were presented cumulatively over 10 days of measurement. A significantly higher urine volume was observed in WT female mice treated with UGN than in male mice (Additional Fig. 1).

**Fig. 5:**
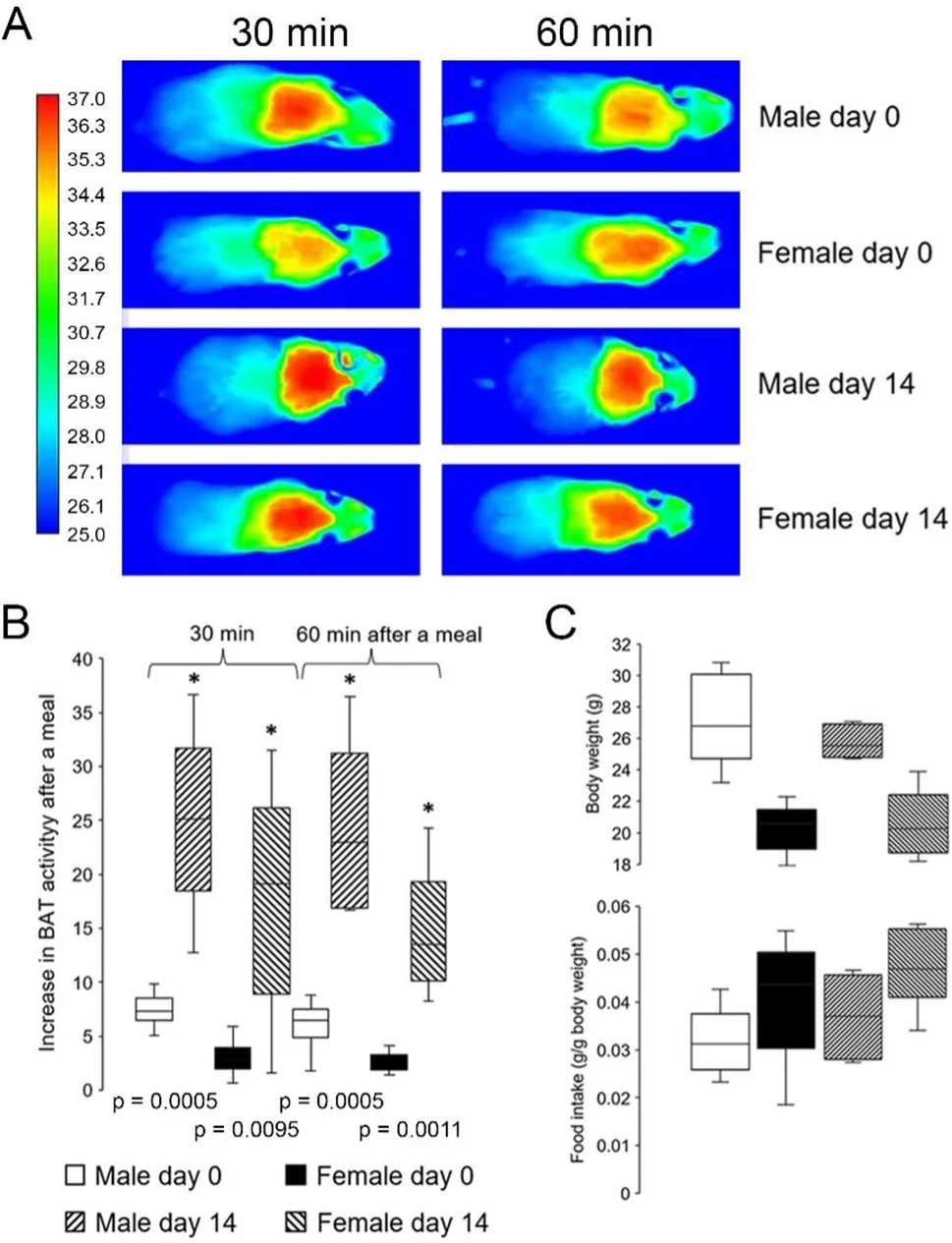
Increased postprandial BAT activity after chronic UGN application in male and female mice in diestrus. For 2 weeks animals received UGN intranasally twice a day (5 µg/animal in 5 µL saline per administration) with final daily UGN dose of 10 µg/animal. Increase in BAT activation after a meal in paired experiments showing results before and after chronic i.n. UGN application (**A** – original thermal images, **B** - summarized results) * *p* < 0.05 compare to starting values at day 0 (Two-Way ANOVA, 30 min: between genders: *p* = 0.03, F = 5.4879, η^2^ = 0.2, before and after UGN application: *p* = 0.000011, F = 34.8789, η^2^ = 0.65, 60 min: between genders: *p* = 0.0052, F = 9.962, η^2^ = 0.34, before and after UGN application: *p* = 1*10^− 6^, F = 48.1719, η^2^ = 0.72, *n* = 5–6). There was no difference in body weight and food intake before and after UGN application (**C**). Results are presented as median with interquartile range. BAT – brown adipose tissue, UGN – uroguanylin

After chronic UGN administration, postprandial BAT activation increased after a meal (Fig. 5A – original thermal images, Fig. 5B- summarized results, paired Student’s t-test), with no statistically significant differences in body weight or food intake before treatment (day 0) and after treatment compared with baseline values obtained before treatment (paired Student’s t-test, Fig. 5C). Females were in diestrus during measurement of postprandial BAT activity. Similar results were obtained in unpaired experiments comparing BAT activation after a meal in animals receiving saline or UGN, again with no difference in body weight or food intake between animals receiving saline or UGN for 14 days (Additional Fig. 2).

### Chronic UGN administration increases BAT volume in male mice

**Fig. 6:**
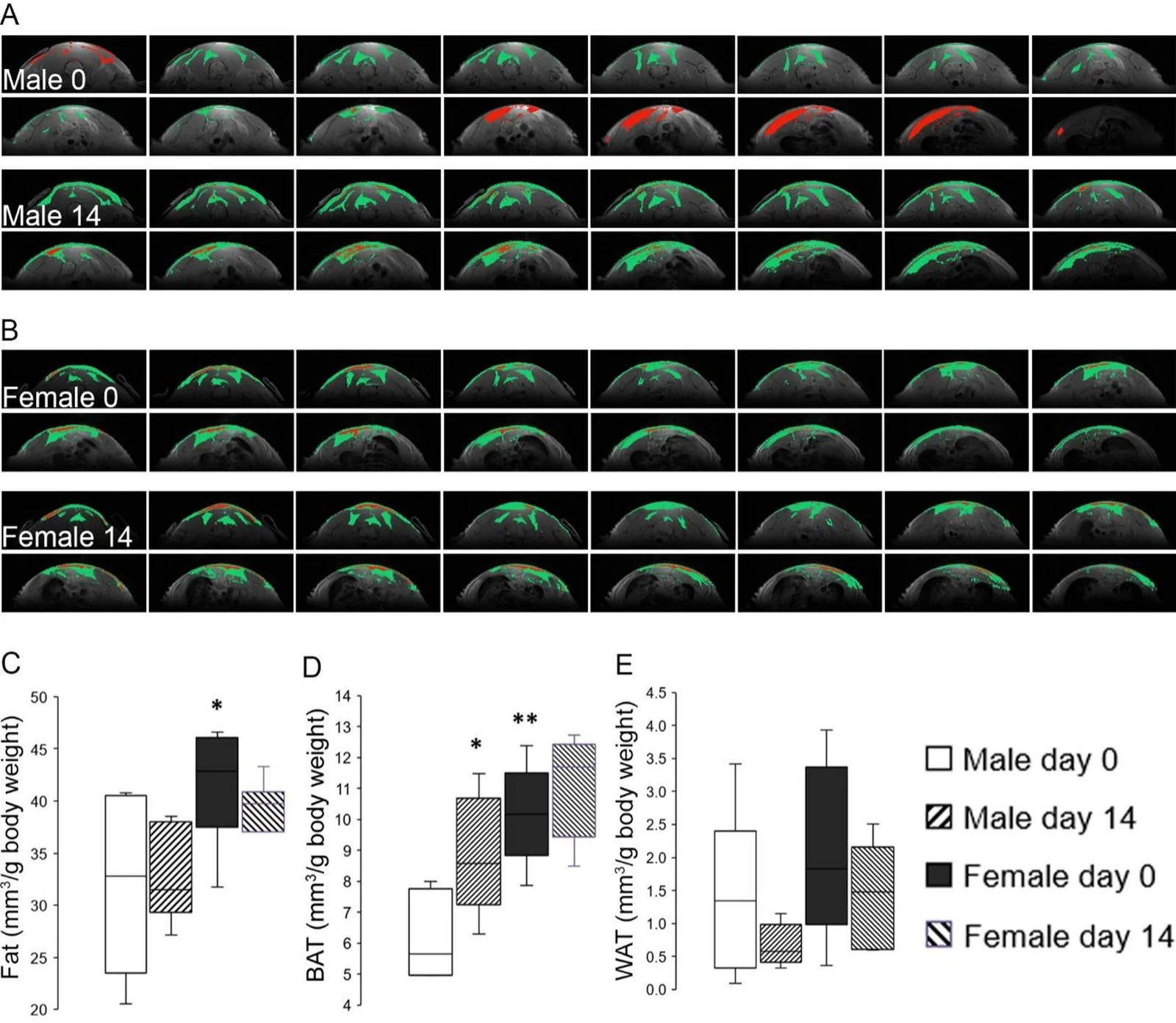
Changes in interscapular fat composition after 14 days of i.n. UGN application. For 2 weeks animals received UGN intranasally twice a day in final daily dose of 10 µg/animal. (**A**) Original MRI scans of male interscapular tissue before (0) and after 14 days of UGN application (14). (**B**) Original MRI scans of female interscapular tissue before and after 14 days of UGN application. The green region denotes brown adipose tissue (BAT) defined by a fat fraction (FF) between 0.2 and 0.7. White adipose tissue (WAT, defined as FF > 0.7) is highlighted in red. (**C**) In female mice before treatment, more fat was detected in interscapular tissue compared to male mice (*p = 0.002; F = 12.83, η^2^ = 0.42, Two Way ANOVA). (**D**) After a UGN treatment BAT volume was only higher in male mice compared to the same animals before treatment (*p = 0.025; F = 5.96, η^2^ = 0.25, Two Way ANOVA). Before treatment, more brown adipose tissue (BAT) was detected in female compared to male mice (** *p* = 0.0004; F = 18.83, η^2^ = 0.51, Two Way ANOVA). (**E**) No statistically significant differences were observed in volume of WAT after UGN treatment. Results are presented as median with interquartile range, *n* = 5–6

To determine possible mechanism of increase in postprandial BAT activation after chronic UGN application, volume of interscapular fat tissues, WAT and BAT was determined by magnet resonance imaging which original scans are presented in Fig. 6A and B. Red colour represents WAT with FF > 0.7 and green colour represents BAT with FF between 0.2 and 0.7.

In the interscapular region, the total volume of adipose tissue was higher in females and was not significantly changed by UGN administration (Fig. 6C). Before treatment, female mice had a greater amount of BAT in the interscapular region than male mice (day 0, Fig. 6D). This amount was not altered by chronic UGN administration. In male mice, however, UGN administration significantly increased BAT volume. Although UGN administration appeared to reduce WAT volume in interscapular adipose tissue, this effect was not statistically significant (sex: *p* = 0.081, F = 3.39, η²=0.15; treatment: *p* = 0.1227, F = 2.609, η²=0.12; two-way ANOVA, *n* = 5–6; Fig. 6E).

**Fig. 7:**
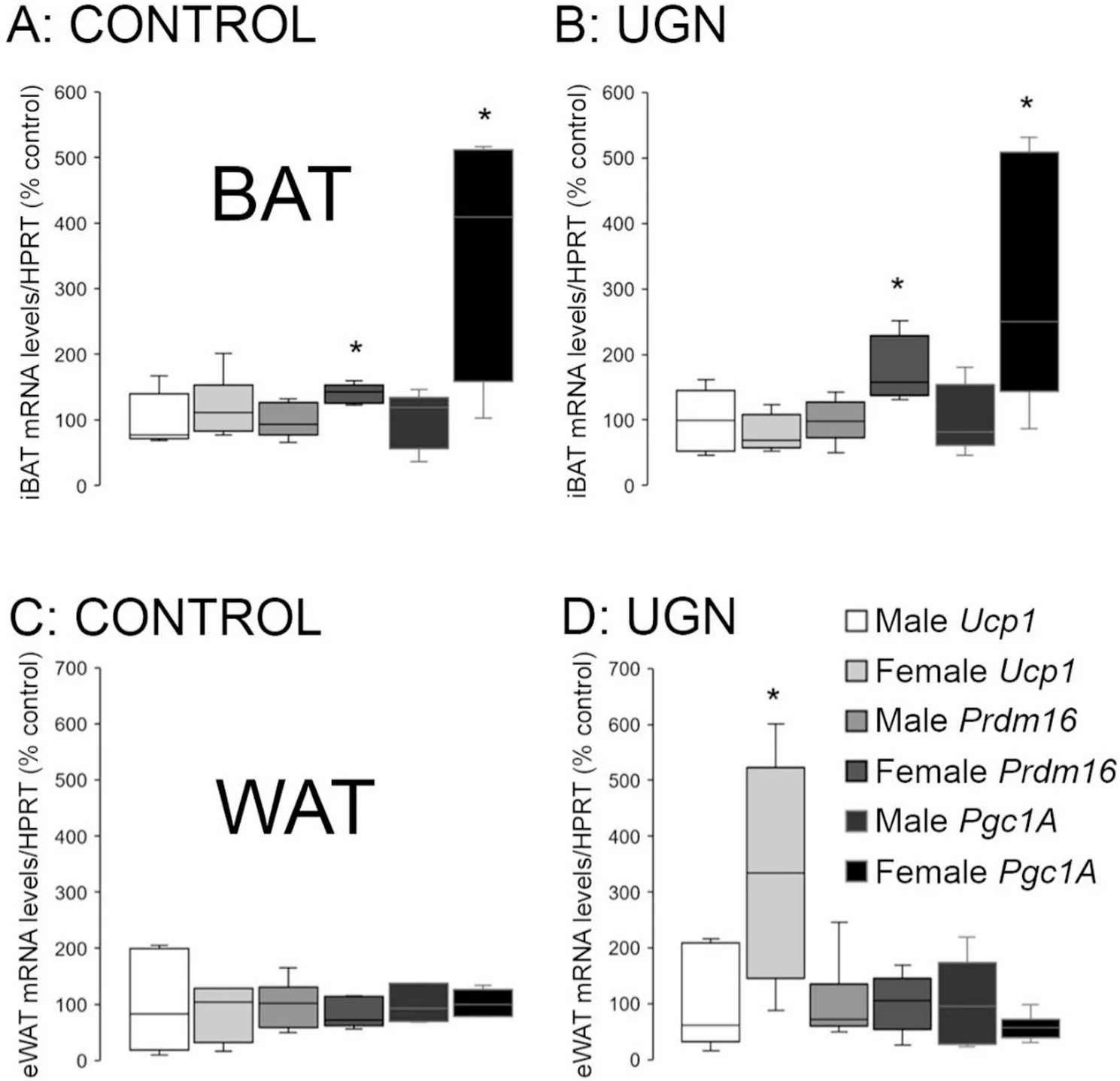
Sex differences in expression of markers of browning in interscapular BAT and epigonadal WAT. (**A**) After 14 days of i.n. saline (control) application in iBAT of female mice in diestrus *Prdm16* and *Pgc1A* were more expressed compared to male mice (*p < 0.05, unpaired Student’s t-test; PRDM16: *p* = 0.0094, t(9) = 3.285, *n* = 5; *Pgc1A*: *p* = 0.0181, t(8) = 2.962, *n* = 5). (**B**) After 14 days of i.n. UGN application, in iBAT of female mice in diestrus PRDM16 and *Pgc1A* were more expressed compared to male mice (*p < 0.05, unpaired Student’s t-test; *Prdm16*: *p* = 0.02, t(8) = 2.884, *n* = 5; *Pgc1A*: *p* = 0.03, t(10) = 2.542, *n* = 6). (**C**) No differences in markers of browning were detected in eWAT after 14 days of i.n. saline (control) application. (**D**) After 14 days of i.n. UGN application, *Ucp1* was more expressed in eWAT of female mice in diestrus (* *p* = 0.05, t(8) = 2,288, *n* = 4–6, unpaired Student’s *t*-test). Results are presented as median with interquartile range. eWAT: epigonadal white adipose tissue; *Hprt*: hypoxanthine-guanine phosphoribosyltransferase; iBAT: interscapular brown adipose tissue; *Pgc1A*: peroxisome proliferator-activated receptor-gamma coactivator 1-alpha; *Prdm16*: positive regulatory domain zinc finger region protein 16; *Ucp1*: uncoupling protein 1; UGN: uroguanylin

Due to no increase in BAT volume upon chronic UGN application only in male mice, to better understand the increase in postprandial BAT activity after UGN treatment in female mice, animals were sacrificed 4 h after a meal, and interscapular BAT and epigonadal WAT were collected. Particular attention was paid to the phase of the estrous cycle in females, which, as during determination of postprandial BAT activity, was diestrus. After 14 days of i.n. saline administration, *Prdm16* and *Pgc1A* were more highly expressed in interscapular BAT (iBAT) of females in diestrus than in male mice (Fig. 7A). Similar results were obtained after 14 days of i.n. UGN administration (Fig. 7B). In epigonadal WAT, only *Ucp1* was more highly expressed in female mice in diestrus than in male mice (*p* = 0.05) after UGN administration (Fig. 7C, D).

### GLP-1 as a regulator of brain proUGN expression

Because postprandial BAT activity declines with age, particularly in females, this part of the study was performed in two groups of animals: young mice (2.2 ± 0.1 months, *n* = 28) and older mice (7.0 ± 0.1 months, *n* = 28). After 4 h of fasting, both groups of male mice and female mice in diestrus were randomly divided into two subgroups receiving either 10 mU insulin or 30 µg i.n. liraglutide (GLP-1 analogue). BAT activity was measured again 30 min later.

**Fig. 8:**
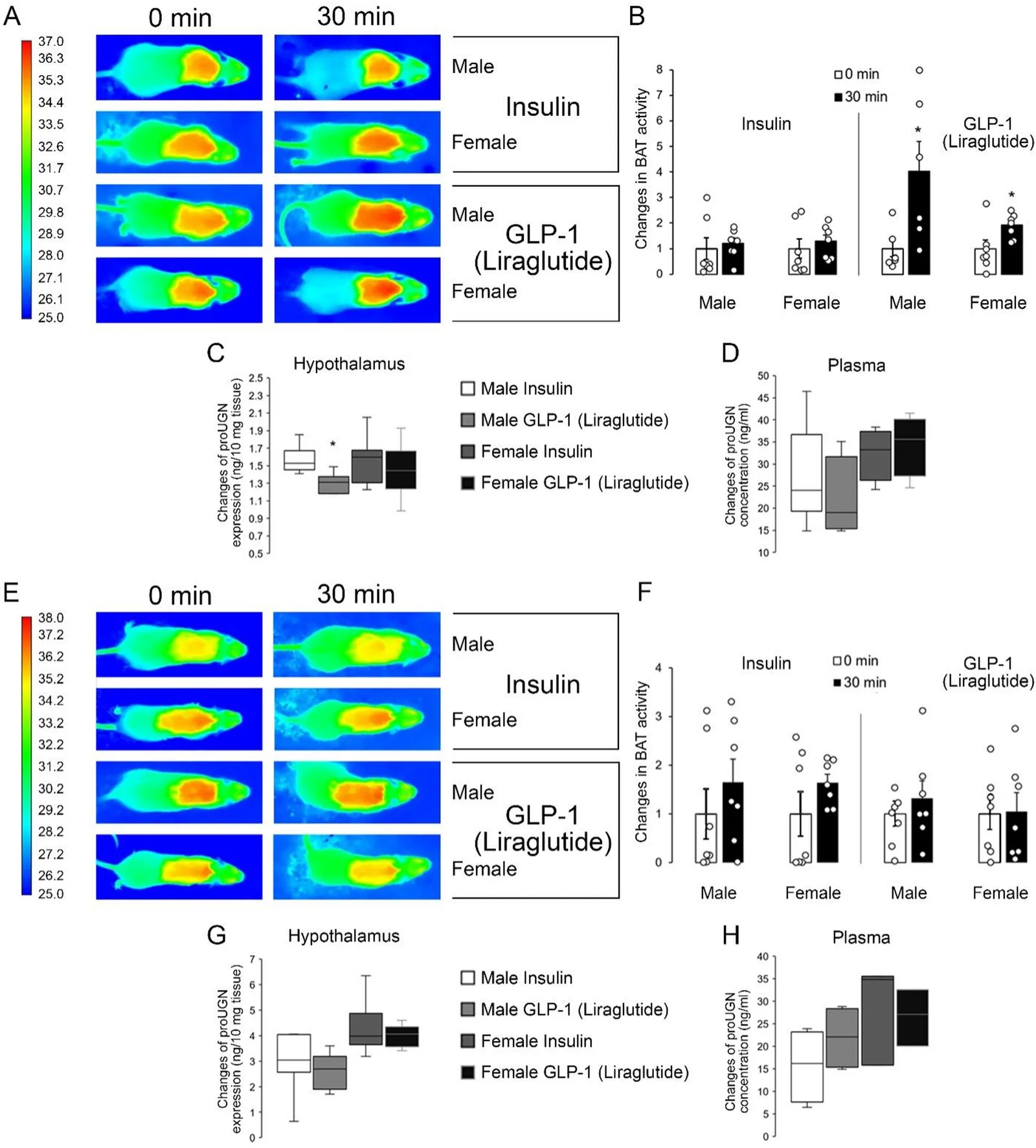
GLP-1 analogue, Liraglutide, changed BAT activity and proUGN expression in hypothalamus in young male mice. GLP-1 analogue, but not insulin, increased BAT activity 30 min after intranasal application in young male mice and female mice in diestrus (**A**-thermal images, **B**-summarized results, *n* = 6–7, * p¸0.05 – compared to starting values, WTM: *p* = 0.0295; t(10) = 2.537; WTF: *p* = 0.0302, t(12) = 2.457, Student’s *t*-test). (**C**) Lower proUGN expression in hypothalamus upon GLP-1 analogue application was observed only in young male mice compared to male animals of the same age upon insulin application (* *p* = 0.0033, t(12) = 3.651, Student’s *t*-test, *n* = 7). (**D**) Similar changes in proUGN levels were not observed in plasma (*n* = 4–6, unpaired One-Way ANOVA, Tukey-Kramer Multiple Comparison test). Neither GLP-1 analogue nor insulin changed BAT activity 30 min after intranasal application in old male mice and female mice in diestrus (**E**-thermal images, **F**-summarized results, *n* = 7, unpaired Student’s t-test). (**G**) No changes in proUGN expression were observed in hypothalamus between GLP-1 analogue or insulin application (*n* = 7, unpaired One-Way ANOVA, Tukey-Kramer Multiple Comparison test). (**H**) In older mice no differences in proUGN concentrations were observed in plasma upon insulin or GLP-1 analogue (*n* = 3–4, Two ANOVA, Tukey-Kramer Multiple Comparison test). Results are presented as median with interquartile range. GLP-1: glucagon-like peptide-1; proUGN: prouroguanylin

In young male mice and female mice in diestrus, the GLP-1 analogue, but not insulin, increased BAT activity 30 min after intranasal administration (Fig. 8A-thermal images, 8B-summerized results). However, the mechanism underlying this increase appeared to differ. The GLP-1 analogue, Liraglutide, decreased proUGN expression in the male hypothalamus compared with insulin, whereas this was not observed in female mice in diestrus (Fig. 8C). The lower hypothalamic proUGN levels after GLP-1 analogue administration were not accompanied by similar changes in plasma proUGN concentration (Fig. 8D). There was no correlation between plasma proUGN concentration and hypothalamic proUGN expression in young male or female mice.

In 7-month-old mice, neither the GLP-1 analogue nor insulin significantly changed BAT activity, and no difference in hypothalamic proUGN expression or plasma concentration was observed between insulin and GLP-1 analogue administration (Fig. 8E-H). However, proUGN levels were higher in the hypothalamus of both groups of older female mice (older mice: insulin: male, 2.96 ± 0.44; female, 4.37 ± 0.40 ng/10 mg tissue; GLP-1 analogue: male, 2.67 ± 0.26; female, 3.98 ± 0.16 ng/10 mg tissue; *n* = 7; *p* = 0.00049, F = 16.233, η²=0.4; two-way ANOVA). Surprisingly, all groups of mice showed significantly higher hypothalamic proUGN expression in older mice than in younger mice (young mice: insulin: male, 1.56 ± 0.06 ng/10 mg tissue; female, 1.56 ± 0.10 ng/10 mg tissue; GLP-1 analogue: male, 1.31 ± 0.04 ng/10 mg tissue; female, 1.47 ± 0.11 ng/10 mg tissue; insulin treatment: *p* = 4*10^− 7^, F = 47.87, η²=0.67; GLP-1 analogue treatment: *p* = 3*10^− 11^, F = 133.12, η²=0.85; *n* = 7; two-way ANOVA).

## Discussion

It is well established that metabolic mechanisms and the occurrence of metabolic diseases differ between men and women and depend on sex hormones and age, with particularly pronounced changes during menopause. BAT activation after cold exposure or after a meal is no exception, and the physiological function of BAT in females is markedly different from that in males [2, 12, 20]. Estrogen is known to increase BAT activity through its action on the hypothalamus [9, 34, 35]. In women, cold-induced thermogenesis is higher than in men, but lower during the luteal phase of the menstrual cycle. Similarly, in 3–5-month-old female mice, cold-induced thermogenesis is higher than in male mice [2]. These measurements were performed by PET-CT using ^18^F-FDG accumulation, which reflects only glucose as an energy source for heat production in BAT. However, no correction for glucose concentration was performed [36]. When assessed by infrared thermal imaging in women aged 20 and 40 years, basal BAT activity and BAT activity after cold exposure were higher during the luteal phase of the menstrual cycle [37]. However, in women aged 21–25 years, Taniguchi et al., using the same method, reported similar BAT activity during cold exposure in the follicular and luteal phases. Unfortunately, those experiments were not paired and were performed during the cold season, when BAT activity may already have been elevated before experiments prior cold exposure [38]. In addition to age, sex, and menstrual cycle phase, cold-induced BAT activation is reduced in participants with obesity [4, 39].

BAT activation after a meal is not readily detectable by PET-CT, probably because blood glucose concentrations are higher than under fasting conditions. Infrared thermal imaging is therefore used to measure postprandial BAT activation. This activation decreases with age and BMI. It is negatively correlated with postprandial blood glucose concentration and positively correlated with DIT. The dependence of DIT on BMI and age is more pronounced in female participants [12]. In 2-month-old female mice, postprandial BAT activation is GC-C-dependent and lower than in male mice. In 8-month-old mice BAT activation after a meal is no longer measurable; however, intranasal UGN administration still increases BAT activity in male mice via brain GC-C, and this activation is negatively correlated with blood glucose concentration. In the present study, in 6-month-old mice intranasal UGN administration (10 µg/mouse) increased BAT activity more than fivefold compared with i.p. administration (50 µg/mouse), suggesting the importance of brain-derived rather than systemic UGN in acute BAT activation, as previously shown for browning [24]. Intranasal administration only slightly activated BAT in female mice in diestrus (luteal phase), whereas no significant BAT activation was observed after i.p. administration, and sex differences were not detected. Neither i.n. nor i.p. administration induced BAT activation in female mice in estrus (follicular phase). However, BAT activation after a meal, when physiological amounts of UGN are released, was UGN-dependent only in male mice. This difference in BAT activation was not due to differences in food intake.

Differences in the physiological role of BAT after a meal and in its age-related changes may result from differences in BAT volume, hormones regulating BAT activity, and their receptors. The fact that postprandial BAT activation is sex- and sex hormone-dependent is not surprising, given that hypothalamic regulation of proopiomelanocortin (POMC) neuron-driven browning differs between male and female mice [40]. The UGN receptor, GC-C, is expressed in POMC neurons of the hypothalamic arcuate nucleus (ARC), which are crucial for the regulation of satiety, BAT activity, and ultimately glucose and body weight homeostasis, all of which depend on sex [2, 41–43]. Furthermore, GC-C expression is regulated by feeding only in male subjects [2, 17, 20]. To further complicate the field, in female mice in diestrus, hypothalamic GC-C expression is higher than in estrus, indicating stronger activation by potential agonists during the diestrus phase of the estrous cycle [20].

Sex differences are observed not only in GC-C expression but also in proUGN expression. ProUGN expression decreased after a meal in the male mouse hypothalamus, possibly due to its processing into UGN, whereas female mice in diestrus had lower proUGN expression under fasting conditions, when GC-C expression is higher than in mice in estrus [20]. Differences in the regulation of hypothalamic proUGN and/or GC-C expression after a meal in male and female mice may explain why postprandial BAT activation is UGN-dependent only in male mice. Although females have a larger BAT volume, UGN does not increase BAT activity to the same extent as in males, possibly due to insufficient hypothalamic UGN availability.

Chronic intranasal UGN administration in male mice increased postprandial BAT activity and BAT volume. In female mice, chronic UGN administration increased postprandial BAT activity but not BAT volume, even though interscapular BAT volume was higher before UGN treatment. In male mice, GC-C is involved in the regulation of BAT markers, including *Pgc1A*, *Prdm16*, and *Ucp1* [2]. In female mice, *Prdm16* and *Pgc1A* expression was higher than in male mice after chronic UGN administration, but also after vehicle administration. These findings are consistent with the study by Takeuchi et al., suggesting that sex differences in BAT activity are due to differences in *Pgc1A* function [44].

Changes in BAT volume and browning markers cannot explain the increased postprandial BAT activation after chronic UGN administration in female mice. One possible mechanism involves systemic effects of intranasally applied UGN binding to GC-C receptors in eWAT [15] of female mice increasing its expression compared to male mice. This result is not surprising given that women have greater browning potential than men [45]. When female WAT expresses more *Ucp1*, other GC-C agonists, such as guanylin, may increase postprandial thermogenesis. The sex-dependent systemic effects of guanylin peptides, however, were not the focus of the present study.

Some of the hormones that increase postprandial BAT activity could regulate hypothalamic proUGN expression and thereby contribute to sex- and age-dependent differences in UGN and BAT function. Leptin and GLP-1 are strong candidates for such a role [43, 46]. Leptin, via GABAergic neurons in the ARC, inhibits neurons in the paraventricular hypothalamic nucleus and thereby activates BAT [47]. However, peak thermogenesis in men occurs 2 h after a meal [48], whereas plasma leptin concentration rises much later, approximately 8 h after a meal [49]. Furthermore, transplantation of BAT into ob/ob mice (leptin KO animals) reduces obesity and improves glucose metabolism [50], suggesting that leptin is not responsible for the regulation of all BAT functions [51].

Although intranasal administration of 160 IU/day insulin affects thermogenesis and metabolism in men but not women [52, 53], in the present study intranasally administered insulin (10 mU, estimated as 20% of the dose used in human studies) did not increase BAT activity in 2- and 7-month-old male mice or female mice in diestrus. As expected, no differences in hypothalamic proUGN expression were observed in either young or old male and female mice.

Another hormone secreted after a meal that activates BAT and increases UCP1 expression via hypothalamic pathways is GLP-1 [46]. Postprandial GLP-1 secretion, particularly 30 min after a meal, is reduced in patients with T2DM [54]. In contrast to insulin, activation of hypothalamic GLP-1 receptors activates BAT [55]. In our study, intranasal administration of a GLP-1 analogue, Liraglutide, to young mice increased BAT activity in male mice and female mice in diestrus, while a statistically significant decrease in hypothalamic proUGN expression was observed only in male mice. Accordingly, there was no difference in BAT activation by GLP-1 in female UGN KO mice (data not shown). Therefore, GLP-1, acting via UGN, may contribute to the age-related decline in BAT function in male subjects. Although the therapeutic effects of GLP-1 analogues in obesity are greater in females, this is probably due to increased satiety rather than increased BAT activity.

Finally, brain UGN and BAT activation appear to be involved in the mechanisms of obesity in humans, but differently in male and female subjects [23]. In the hypothalamus of male subjects with normal body weight, proUGN expression was higher and negatively correlated with age. This correlation was lost in overweight individuals. In the hypothalamus of female subjects, no statistically significant correlation between age and proUGN expression was observed. Increased body weight also affected proUGN expression in other brain regions. ProUGN expression was lower in BA10 and BA11, but only in male overweight subjects, whereas proUGN expression was higher in the cerebellar cortex of overweight females than in females with normal body weight or overweight male subjects. The physiological and pathophysiological significance of these findings remains to be determined in future studies.

### Limitations of the study

In this study, experiments were performed in 6–7-month-old mice in reproductive stage of life whereas deceased persons with the average age of 53 years for males and 64 years for females, the latter being within the menopausal age range. Since ageing is a major contributor to changes in GC-C and UGN expression, comparisons between mouse and human results should be interpreted with caution. These findings also highlight the need to carefully consider the age of the studied subjects.

Sex differences in the relationship between postprandial proUGN brain regulation and the postprandial increase in GLP-1 need further confirmation, especially in human populations with obesity or T2DM. This is currently being investigated as the main topic of ongoing studies in our laboratory.

### Perspectives and significance

New therapeutic approach in obesity and T2DM is activation of BAT to improve postprandial glucose regulation and increase glucose expenditure. In mice, postprandial regulation of BAT activity may be driven by regulation of proUGN expression only in the male hypothalamus. Similarly, in male, but not female mice, intranasally applied GLP-1 analogue, Liraglutide, decreased hypothalamic proUGN expression followed by an increase in BAT activity. In human overweight subjects usually accompanied by decreased BAT activity, lower proUGN expression in hypothalamus and parts of PFC is shown only for male subjects suggesting different roles of brain UGN in male and female human subjects. In future studies we plane to investigate sex differences of GLP-1 and connection to brain UGN in human population. The development of brain BAT activators and rejuvenation of BAT as a therapy for obesity and T2DM must take sex into account.

## Conclusions

The molecular mechanism of obesity, especially expression of regulatory hormones and their receptors is not well investigated in the male and female human brain. In our study, we showed that in obesity the role of brain UGN differs between the male and female human brain. In hypothalamus of obese male subject there is less proUGN leading to lower activation of UGN when compared to subjects with normal body weight. Accordingly, in laboratory animals, brain UGN and its regulation by GLP-1 followed by increase in BAT activity has important role only in male mice. Therefore, it is not surprising that chronic application of UGN increase BAT volume again only in male mice. Summarized findings are shown in Table 1. When therapy applied in obesity subjects or patients with T2DM include hormones which activates and rejuvenate BAT, sex difference should be take into account.

**Table 1:** Summarized sex differences in BAT activation and proUGN expression in the brain

Effect	Male	Female
Different proUGN expression in human hypothalamus, BA10 and 11 in obesity	Yes	No
Postprandial BAT activation by UGN	Yes	No
Decrease postprandial hypothalamic proUGN expression	Yes	No
Increased BAT activation upon i.n. UGN application	Yes	Estrus – No Diestrus – Yes (low)
Increase in BAT activity by chronic i.n. UGN application	Yes	Yes
- by increased BAT volume	Yes	No
Increase in BAT activity upon i.n. GLP-1 application	Yes	Yes (low)
- by changes in hypothalamic proUGN expression	Yes	No

## Supplementary Information

Below is the link to the electronic supplementary material.

Supplementary Material 1

## Data Availability

All data reported in this study is available from the corresponding author Aleksandra Dugandžić (aleksandra.dugandzic@mef.hr).

## Abbreviations

^18^F-FDG: ^18^F Fluorodeoxyglucose
ANOVA: Analysis of Variance
ARC: Arcuate hypothalamic nucleus
BA: Brodmann area
BAT: Brown adipose tissue
iBAT: Interscapular brown adipose tissue
BMI: Body mass index
DE: Diestrus
DIT: Diet induced thermogenesis
E: Estrus
ELISA: Enzyme-Linked Immunosorbent Assay
F: Fasting
GC-C: Guanylate cyclase C
GLP-1: Glucagon-like peptide-1
HPRT: Hypoxanthine-guanine phosphoribosyltransferase
i.n.: Intranasal
ins: Insulin
i.p.: Intraperitoneral
M: Medial
MRI: Magnetic Resonance Imaging
O: Orbital
PET-CT: Positron Emission Tomography/Computed Tomography
PFC: Prefrontal cortex
Pgc1A: Peroxisome proliferator-activated receptor-gamma coactivator 1-alpha
POMC: Proopiomelanocortin
Prdm16: Positive regulatory domain zinc finger region protein 16
qPCR: Quantitative polymerase chain reaction
T2DM: Type 2 diabetes mellitus
UCP1: Uncoupling protein 1
UGN: Uroguanylin and prouroguanylin (proUGN)
UGN KO: UGN knock out littermates
WAT: White adipose tissue and epigonadal WAT (eWAT)
WTF: Wild type female mice
WTM: Wild type male mice
