# Supplementary material for: Different roles of brain uroguanylin and brown adipose tissue in males and females
Source: Biol Sex Differ. 2026 Aug 11;17:177. doi: 10.1186/s13293-026-00965-y (PMC13746522; doi:10.1186/s13293-026-00965-y)
Supplement: Supplementary file 1 [file 13293_2026_965_MOESM1_ESM.docx]

**Different roles of brain uroguanylin and brown adipose tissue in males and females**

Nikola Habek^1-3^, Martina Ratko^1,2*^, Milan Kordić^4^, Marina Dobrivojević Radmilović^1^, Siniša Škokić^1^, Vladiana Crljen^1-3^, Martina Tkalčić^5^, Anton Mažuranić^5^, Pero Bubalo^5^, Petar Škavić^5^, Aleksandra Dugandžić^1-3 *^

Table 1: Brian samples were taken from following deceased participants.

| No. | M/F | Body weight | Age (years) | Cause of death |
| --- | --- | --- | --- | --- |
| 1 | Female | Normal | 88 | Heart failure |
| 2 | Female | Normal | 96 | Heart failure |
| 3 | Female | Obese | 64 | Heart failure |
| 4 | Male | Obese | 57 | Heart failure |
| 5 | Female | Obese | 70 | Drowning |
| 6 | Male | Obese | 59 | Heart failure |
| 7 | Male | Obese | 34 | Electric shock |
| 8 | Male | Normal | 39 | Acute poisoning |
| 9 | Male | Normal | 67 | Thromboembolism of pulmonary arteries |
| 10 | Male | Normal | 55 | Heart failure |
| 11 | Male | Obese | 48 | Choking on a bite of food |
| 12 | Male | Obese | 52 | Heart failure |
| 13 | Male | Obese | 63 | Heart failure |
| 14 | Female | Normal | 18 | Heart failure |
| 15 | Male | Normal | 61 | Acute poisoning |
| 16 | Female | Obese | 47 | Thromboembolism of pulmonary arteries |
| 17 | Male | Normal | 91 | Suicide |
| 18 | Male | Normal | 41 | Suicide |
| 19 | Male | Obese | 41 | Heart failure |
| 20 | Female | Normal | 60 | Brain haemorrhage |
| 21 | Male | Normal | 50 | Suicide |
| 22 | Male | Normal | 31 | Hear failure and pneumonia |
| 23 | Male | Obese | 74 | Choking on a bite of food |
| 24 | Male | Normal | 40 | Acute poisoning |
| 25 | Male | Normal | 67 | Internal bleeding |
| 26 | Male | Normal | 66 | Heart failure |
| 27 | Female | Normal | 57 | Heart failure |
| 28 | Female | Obese | 67 | Heart failure |
| 29 | Female | Obese | 70 | Fracture of the 2^nd^ cervical vertebra |
| 30 | Female | Normal | 89 | Severe atherosclerosis, multiple injuries |
| 31 | Male | Obese | 60 | Cardiac tamponade |
| 32 | Female | Normal | 78 | Massive haemorrhages |
| 33 | Male | Obese | 31 | Suicide |
| 34 | Female | Obese | 68 | Heart failure |

**Figure 1.** Sex after 14 days of intranasal saline or uroguanylin administration. Results are presented as mean ± SEM. *p < 0.05, statistically significant difference compared with male mice (Student’s t-test, n = 6). UGN, uroguanylin.

**Figure 2.** Increased postprandial BAT activity after chronic UGN administration in females in diestrus and males. BAT activation after a meal was increased in animals after 14 days of i.n. UGN administration compared with animals after 14 days of i.n. saline administration (A). No differences in body weight or food intake were observed between saline- and UGN-treated animals (B). *p < 0.05, unpaired Student’s t-test, n = 5–6. Results are presented as median with interquartile range. BAT, brown adipose tissue; UGN, uroguanylin.

**METHODS**

*Metabolic cages*

To determine the chronic effects of UGN, postprandial BAT activation was examined in male and female WT mice and UGN KO littermates housed in metabolic cages. Twelve male and twelve female mice of both genotypes were randomly assigned to two groups receiving either saline (vehicle) or UGN (10 μg/animal). Solutions were administered twice daily (5 μL per administration), in the morning (8 a.m.) and in the afternoon (4 p.m.), for 2 weeks. After four days of acclimatization, food and water intake, as well as fecal and urine output, were measured. Urine was collected under a thin layer of mineral oil to prevent evaporation.
